# Social trust and the location choices of foreign firms in China–The moderating role of formal institution and cultural distance

**DOI:** 10.3389/fpsyg.2023.1061813

**Published:** 2023-02-23

**Authors:** Huiyun Shen, Changjun Yi, Jie Yu, Jin Gou

**Affiliations:** ^1^School of Business Administration, Huaqiao University, Quanzhou, China; ^2^College of Computer Science and Technology, Huaqiao University, Xiamen, China

**Keywords:** social trust, location choice, informal institution, formal institution, cultural distance, conditional logit model

## Abstract

The institutional environment has a significant impact on the location of overseas investments by multinational enterprises (MNEs). However, there remain two research gaps. First, fewer studies focused on the impact of subnational regional institutions on the location choices of MNEs. Second, study on informal institutions has been more limited. This study investigates the effect of the informal institution (social trust) in the Chinese subnational region on the location choices of foreign firms and the mechanism of its role. Using the sample of foreign firms’ location choices in China from 2008 to 2020 in Orbis Global Enterprise Database, this study finds that social trust positively related to the location choices of foreign firms in subnational regions. Our results also show that this positive effect is contingent on the formal institution and the cultural distance between home and host country. When the formal institution is strong and the cultural distance between home and host country is high, social trust has a more significant positive impact on the location choices of foreign firms in subnational regions. Besides, the results show that cost advantage, information advantage and innovation advantage are important mechanisms for social trust to influence foreign firms’ location choices in subnational regions. This study is important for understanding the role of subnational regional informal institutions in influencing strategic decisions of MNEs. At the same time, it has certain guiding significance for governments in attracting foreign direct investment and for multinational enterprises in selecting suitable overseas investment locations.

## 1. Introduction

For Multinational Enterprises (MNEs), choosing a right location has a profound impact on the efficiency and performance of their overseas investments (Bartik, 1985; Jain et al., 2016). Therefore, the choice of location is central to MNEs’ overseas investment strategy (Dunning, 2009; Buckley, 2016). However, compared to other strategic decisions of MNEs, only a few scholars have focused on location choice, and the findings are inconsistent or even contradictory (Romero-Martínez et al., 2018), so scholars have called for more research on location choices of MNEs (Hitt et al., 2016; Nielsen et al., 2017).

Previous studies, based on theories such as transaction cost theory, eclectic paradigm and internationalization process model, explored the impact of firms’ own investment motives (Dunning, 2009), internationalization experience (Johanson and Vahlne, 1977), host country infrastructure development (Cheng and Kwan, 2000), labor quality (Gao, 2005), market size (Birkinshaw et al., 2006), and natural resources (Yao et al., 2017) on MNEs’ location choices. In recent years, more and more scholars have focused on the importance of institutions in the choice of location for overseas investment, rather than studying firm behavior in a “vacuum” (Dau et al., 2022). Institutions are the formal and informal “rules of the game in a society” (North, 1990), which include formal and informal institutions. Foreign investors must follow and conform to the “rules of the game” of the host country in order to ensure the effective operation and profit growth of their businesses. It has been shown that countries or regions with the “right” institutions can reduce transaction costs and uncertainty, thus achieving sustainable economic growth (Kim and Aguilera, 2016). In contrast, a weak institution increases the negotiation and implementation costs of overseas investments, thus discouraging potential transactions (Meyer, 2001). Therefore, research on the influence of the institutional environment on the MNEs’ overseas investment strategy has become an important part in the field of international business (Xu and Shenkar, 2002).

While some of the literature has examined the influence of institutions on the location choice of MNEs investing abroad (Beazer and Blake, 2018; Davies and Killeen, 2018; Noon et al., 2019), two research gaps remain. First, in terms of the unit of analysis, previous scholars have mainly studied country-level institutions on the location choices of MNEs, while fewer studies focused on the impact of subnational regional institutions on the location choices of MNEs (Nielsen et al., 2017). Indeed, as Chan et al. (2010) suggested, subnational regions are important when considering the final location decisions of MNEs in host countries. Because the inconsistent formulation and implementation of governmental policies and rules (Chan et al., 2010), and the unique cultural traditions and/or social values that are different from region to region in a host country (Chan et al., 2010), and have an impact on firms’ transaction costs and operational uncertainty. Since the final location chosen by MNEs for their subsidiaries in foreign markets are specific subnational region rather than a single host country, intra-country heterogeneity at the subnational level is likely to be at least as important a determinant for the FDI location choice as inter-country heterogeneity at the national level (McCann and Mudambi, 2005).

Second, although some scholars have studied the relationship between institutions in subnational regions of the host country and the location choice of FDI, as Lu et al. (2018) and Dau et al. (2022) have pointed, the bulk of prior research has focused on formal institutions, such as the level of corruption (Brada et al., 2012), host country governance (Louie and Rousslang, 2007) or tax rates (Davies and Killeen, 2018). In contrast, study on informal institutions has been more limited (Sartor and Beamish, 2014). Actually, even in countries with strong formal institutions, the number of informal institutions is more than formal institutions (North, 1990), and some of the most striking differences between countries are often caused by informal institutions (Bruton et al., 2009). In recent years, more and more scholars have called for more research on informal institutions (e.g., Lu et al., 2018; Brockman et al., 2020; Dau et al., 2022), to increase understanding of how informal institutions affect firms’ internationalization strategies and performance.

This study attempts to fill the above two gaps. Firstly, we investigate the effect of the informal institution (social trust) in the Chinese subnational region on the location choices of foreign firms and the mechanism of its role. Social trust, as an expectation of the future, is a core component of informal institutions that shapes the rules of economic transactions and have an important impact on the behavior of economic agents (Deutsch, 1958). For example, Guiso et al. (2009) found that social trust is an important factor influencing OFDI and trade between countries. In a society with high level social trust, members in such regions are more willing to trust others and expect to reduce transaction costs and improve business efficiency by cooperating with others (Lu et al., 2018). Therefore, trust mechanism that reduce costs and uncertainty may become a new perspective to explain the location choices of MNEs. Second, we delve into how subnational formal institutions moderate the strength of social trust influence on FDI locational choices. Formal institutions include a set of laws and regulations and a regulatory force that governs the establishment and enforcement of contracts (North, 1990). Because the interpretation and appreciation of the regulations and laws are significantly different across regions and so are their implementation in a country, leading to differences in the development of formal institutions at subnational level in many countries (Chan et al., 2010; Ma et al., 2013). More importantly, well-established formal institutions provide a stable external guarantee for business operations and can increase and complement the effectiveness of informal constraints (North, 1990). Finally, we delve into how cultural distance between home and host countries moderate the strength of social trust influence on FDI locational choices. Cultural distance, considered an informal institution that reflects cultural differences between home and host countries (Bailey, 2018). Although it is not the same as subnational formal institutions, or even a subnational level factor, we include it here for three reasons. First, it is cited in a large number of articles as a deterrent to FDI when the cultural distance is high. Second, it is often regarded as an informal institution that influences the understanding of “rules of the game” in the host country. Finally, without taking this into account during location decision-making can result in significant costs to MNEs.

We use the data of foreign firms in China from 2008 to 2020 in Orbis Global Enterprise Database to tackle these unexplored research questions for the following two reasons. Firstly, China presents an ideal place for our investigation, since there are many provinces in China and these provinces differ significantly in terms of social trust and formal institutions (Jin et al., 2016; Lu et al., 2018), which allows us to examine the effects of institutions at the subnational level on subsidiary location choice. Secondly, in recent years, China has become one of the largest recipient countries of FDI in the world, with many foreign firms operating in different subnational locations in China. Therefore, China provides us with the meaningful and significant number of foreign subsidiaries of MNEs needed for our empirical analyses. We find that social trust, through cost advantages, information advantages and innovation mechanisms, helping foreign firms reduce their investment uncertainty and costs in China, and ultimately promoting the location choices of foreign firms. Moreover, we find the effect of social trust in promoting the location choices of foreign firms is greater when the formal institution is strong and the cultural distance between home country of foreign firms and China is high.

This study contributes to the literature in several aspects. Firstly, different from previous studies that have mainly focused on country-level institutional variations (Jin et al., 2016; Brockman et al., 2020; Gaur et al., 2022), this research studies the strategic implications of institutional complexities within a host country. This study is important for understanding the role of subnational regional informal institutions in influencing strategic decisions of MNEs. Specifically, we respond to the call for more research on informal institutions (Brockman et al., 2020; Dau et al., 2022) and expand the literature on institutions by examining the effect of subnational regional informal institutions (an important yet under-studied area) on the location choices of foreign firms and by revealing the mechanisms of the effect. Most prior studies focus on the effect of formal institutions (Young et al., 2018), and we find that social trust in subnational regions influence the location choices of foreign firms through information advantages, cost advantages, and innovation advantages. Thus, our work compensates for the lack of social perspective in the existing literature due to the neglect of the social “embeddedness” of foreign direct investment and contributes to a systematic understanding of the economic effects of social trust as a “soft environment”. Further, it accumulates new knowledge for the interdisciplinary study of informal institutions and location choice.

Secondly, our study enriches the interactive effect of informal institutions and formal institutions. In recent years, although an increasing number of scholars have focused on the impact of institutional factors on international business issues, research has mainly concentrated on the independent impact of specific institutional factors and less on the interactions between different institutions. Formal and informal institutions are interdependent, exist in layers of society (Williamson, 2000), and function in concert with each other (North, 1990). Therefore, the effect of informal institutional must be studied together with other formal institutional factors (Beugelsdijk et al., 2018). We find that strong formal institutions reinforce the effect of social trust on promoting the location choices of foreign firms, suggesting that formal institutions help facilitate the role of informal institutions (North, 1990).

Third, this study makes an important contribution to social trust research. Specifically, we explore the boundaries of the role of social trust in influencing the location choices of foreign firms from the perspective of cultural differences between countries. Distance between countries, especially cultural distance, is considered to be a significant obstacle of overseas investment by MNEs (Bailey and Li, 2015; Liu et al., 2022). We find that social trust can help MNEs overcome the possible negative effects of cultural distance. This result highlights the important role of social trust in helping MNEs reduce the uncertainty and costs of overseas investments, and enriches the social trust research.

Finally, Limited by the unavailability of firm-level foreign direct investment data, most studies on foreign direct investment in China only include city-level or regional-level (Du et al., 2012). We obtained complete foreign firm-level data for China using the Orbis Global Enterprise Database, which provides a more complete record of information related to Chinese foreign firms and their parent companies before 2021 and can effectively paint a complete picture of foreign direct investment in China. What’s more, using firm-level data, we can virtually minimize the concern for endogeneity (including reverse causality) issues in econometric analysis (Du et al., 2012).

The rest of the article is structured as follows: Section “2. Literature review” provides a literature review. Section “3. Hypothesis development” presents the research hypotheses. Section “4. Research design” describes the sample, variables and method. Section “5. Results” reports the empirical results, robustness tests, and the analysis of effect mechanisms. Section “6. Conclusion and discussions” presents the conclusion and discussions.

## 2. Literature review

### 2.1. Institutional theory and location choice of foreign firms

Based on institutional economics (North, 1990) and sociology (Scott, 1995), institutional theory has been advanced as a powerful and frequently applied theoretical approaches for explaining business strategy, and MNE international behavior in particular (e.g., Nielsen et al., 2017; Gaur et al., 2022). There is general consensus that institutions (whether national or sub-national) shape the nature of business by providing the constraints and opportunities under which economic activity occurs. Thus, the nature and quality of institutions in host country influence the location choice of FDI in various ways. In view of this, this study analyzes the effect of subnational regional institutions (social trust) on FDI location choice in China from both theoretical and empirical perspectives based on institutional theory.

According to institutional theory, Institutions are defined as “rules of the games,” both formal (e.g., laws and regulations) and informal (e.g., social trust and norms), that construct the social relationships, political, and economic within a country or society (North, 1990; Scott, 1995). The institutions of a country (or region) affect the profitability of firms embedded in it by influencing the transaction and transformation costs of production (North, 1990). Institutional theory suggests that in order to survive, firms must conform to the values and rules prevailing in their environment (DiMaggio and Powell, 2000). A society with the “right” set of institutions minimizes transaction costs and facilitates more complex exchanges between social participants. Thus, many studies point out that institutional play a key role in influencing the FDI location choices (Bailey, 2018). Specifically, a highly developed institutional environment creates transparency in economic transactions and increases the efficiency of market-based transactions and resource allocation by lowering transaction costs and reducing uncertainty, thereby attracting FDI. In contrast, in regions with weak institutions, firms face high levels of uncertainty and high costs of doing business, which adversely affects the profitability of MNEs’ subsidiaries and thus discourages the attraction of FDI.

### 2.2. Subnational location choice for FDI

For decades, many scholars have been interested in exploring the main factors that influence the location choice for FDI (Yao and Li, 2016; Bailey, 2018; Gao et al., 2019). Traditionally, scholars focused on economic factors such as labor costs, market size, infrastructure, and others as the important factors in influencing a host country’s ability to attract or deter FDI (Dunning, 1980; Grosse and Trevino, 1996). In the 1990s, following the influential work of North (1990), IB researchers began to pay more attention on the influence of institutions on the location choice of FDI (Loree and Guisinger, 1995; Globerman and Shapiro, 2003).

Mostly, this research has taken the country as the unit of analysis to understand the effect of institutions on FDI location choice. As the institutional differences between countries are prominent, it is crucial to study institutional factors when analyzing the firms’ strategic decisions in FDI location choice (Gao et al., 2019). For example, factors such as political stability rule of law and democratic institutions attracting FDI (e.g., Loree and Guisinger, 1995; Globerman and Shapiro, 2003), and factors such as cultural distance, tax policies and corruption (e.g., Loree and Guisinger, 1995; Habib and Zurawicki, 2002; Globerman and Shapiro, 2003) deterring FDI.

In addition, while the majority of studies using institutional perspectives conceptualize institutions at country level, it has been recognized that institutional differences across regions within a country may also affect the attractiveness to foreign investors (Nielsen et al., 2017). When foreign companies operate in an institutional context different from their home countries, they need to decide not only which country to enter, but also in which specific location in that country to invest (Du et al., 2008). Their choice to operate in a specific subnational region is expected to affect the profitability and survival of the subsidiary. This is especially true when the host country is an emerging economy like China, which is culturally and ethnically diverse and has a great deal of domestic heterogeneity in its informal institutions (Li et al., 2018). In summary, social institutions, political and economic vary from region to region within the host country. Such differences in subnational institutions provide challenges and opportunities for foreign firms and thus affect FDI location choices. That is, while the more commonly studied country-level effects are important, more attention needs to be paid to subnational effects on business strategy (Falaster and Ferreira, 2020). In particular, institutional factors may be specific to subnational regions and may affect FDI location choice.

Moreover, while some scholars have studied the impact of sub-national regional institutions on FDI location choice by influencing transaction costs and uncertainty (Du et al., 2012; Falaster and Ferreira, 2020). However, as informal institutions are more difficult to conceptualize and measure, the existing literature has mostly focused on the impact of formal institutions such as subnational marketization (Yang, 2018), contract enforcement (Du et al., 2012), and intellectual property protection (Du et al., 2012) on FDI location choice, while research on informal institutions is relatively limited. This has led to an unbalanced development of the institutional base view, which is not conducive to a comprehensive understanding of the relationship between sub-national institutions and firms’ FDI location choices.

Social trust is a core component of informal institutions, and its essence consists of unwritten social norms of behavior. Arrow (1972) pointed out that “almost every business transaction has an element of trust in itself”, and this idea has led to a large number of related studies. In the field of international business, scholars have mainly explored the impact of social trust on firms’ internationalization strategies and performance at the national or regional level. At the country level, Guiso et al. (2009) earlier found that differences in social trust between countries are an important factor affecting OFDI and trade flows. Brockman et al. (2020) studied the impact of social trust on international contracting and found that the higher the social trust in the bond issuer’s country, the less covenants creditors impose on the bond issuer. Gaur et al. (2022) studied the impact of the interaction of social trust and formal institutions in the host country on the expatriate strategy of subsidiaries based on a corporate governance perspective, and found that high levels of social trust and high-quality formal institutions helped to constrain opportunistic behavior, which in turn reduced the share of expatriates in subsidiaries. At the regional level, Lu et al. (2018) explored the impact of social trust on the performance of foreign subsidiaries in Chinese provinces and found that social trust has a positive impact on subsidiary performance by reducing outsider liability. It can be found that the above-mentioned studies are similar to this paper. They all focus on the role of social trust when exploring the influence of informal institutions on firms’ internationalization strategies and performance. However, the above-mentioned articles do not cover the important internationalization strategy decision of FDI location choice, which leaves an academic gap in the study of this paper. Based on this literature, this paper argues that the location choice of foreign firms is also influenced by subnational institutions (social trust in different regions of the host country).

In summary, institutional theory provides an analytical perspective to explain the role of subnational social trust in FDI locational choice. However, the existing literature remains underdeveloped. First, the existing literature mostly focuses on the impact of country-level institutions or institutional differences on FDI location choice. Second, a few scholars have started to study the impact of subnational institutions on firms’ FDI location choice, but they mainly focus on the role of formal institutions and pay insufficient attention to informal institutions. Based on institutional theory, we seek to reveal the impact of subnational social trust on FDI location choice, and examine the mechanisms of action and their boundary effects in depth. This paper makes up for the lack of attention to subnational informal institutions affecting FDI location choice in existing studies, and enriches the studies related to subnational informal institutions and location choice.

## 3. Hypothesis development

### 3.1. Social trust and location choice of foreign firms

As a core component of the informal institution, social trust shapes the basis of economic transactions and has an important effect on the location choices of foreign firms. First, social trust facilitates the flow of information by influencing the way members of society share and process information, reducing the information asymmetry that foreign firms face when investing overseas, and thus facilitating foreign firms’ location choices. Limited by the lack of knowledge about the laws, markets and cultures of various regions in China, foreign firms have serious information asymmetry, which increases the uncertainty and costs. Social trust helps to promote the willingness of regional members to share information and resources with foreign firms, and it also enhances the authenticity and reliability of information (Jin et al., 2016; Jha, 2019). Therefore, in a society with high level social trust, foreign firms are more likely to obtain truthful and reliable market information and thus increase their understanding of the local business environment, which can effectively alleviate the information asymmetry problem. On the contrary, in regions with low level social trust, foreign firms have difficulties in obtaining information from local stakeholders (Lu et al., 2018).

Second, social trust reduces the transaction costs of foreign firms by making it less difficult for them to gain legitimacy, which eventually facilitates the location choices of foreign firms. Due to the liability of outsider in a region, foreign firms tend to be regarded as out-group members by local stakeholders when they enter host country. As a result, it is more difficult for foreign firms to obtain stakeholder support and tap into the host country’s business network, making it more difficult and costly for foreign firms to gain legitimacy, which eventually hinders their success. Social trust, a major dimension of social capital, enables members in a region to establish common expectations of honest and normative behavior (Knack and Keefer, 1997), which both helps to reduce the complexity of communicative interactions between economic agents (Bjørnskov, 2011; Kim and Li, 2014) and promotes the establishment and maintenance of cooperative relationships between strangers (Porta et al., 1997; Constantin et al., 2013). In a society with high level social trust, members in such region are less likely to “divide the world into friends and enemies” or to interact only among a small circle of familiar ones (Fukuyama, 2001). In such cases, local firms are more willing to accept outsiders in their local networks and share resources and opportunities with outsiders (Lu et al., 2018), and it is easier for foreign firms to develop social and business relationships (Liu et al., 2022). Thus, in regions with higher social trust, foreign firms have higher legitimacy and are able to reach cooperation and share opportunities with stakeholders at a lower cost. In addition, social trust increases the costs of corporate violations through a system of social supervision and sanctions, which subsequently discourages opportunistic behavior and reduces uncertainty and costs for foreign firms (Toshio and Midori, 1994).

Finally, social trust promotes the location choices of foreign firms by enhancing the innovation efficiency in subnational region. Social trust is a lubricant for knowledge diffusion and sharing. The higher the degree of mutual trust in the cooperation process, the higher the frequency of information exchange and sharing, which eventually leads to higher innovation efficiency and output in such region. Quinn (1979) argue that trust can stimulate innovation within and between organizations by reducing the need for monitoring and control mechanisms, and enhancing idea generation through interactions between individuals. Moreover, Doh and Acs (2010) argue that trust can reduce transaction and monitoring costs, encourage individuals in society to cooperate and share resources such as information, skills and knowledge, thus making it possible for a society to promote innovation. Based on the foregoing we propose:

Hypothesis 1: Social trust is positively related to the location choices of foreign firms in subnational regions.

The above analysis suggests that social trust ultimately facilitates the location choices of foreign firms by promoting information flow, reducing foreign firms’ transaction costs, and enhancing innovation efficiency. At the same time, previous studies point out that formal institutions and cultural distance between home and host country are also important factors affecting the uncertainty and costs of overseas investment (Diego et al., 2011; Du et al., 2012; López-Duarte and Vidal-Suárez, 2013; Kunčič and Jaklič, 2014; Kapas, 2020), so they may be important boundary conditions for social trust to influence foreign firms’ investment location choices. Next, we further discuss the moderating role of formal institutions and cultural distance between home and host country.

### 3.2. The moderating role of formal institution

Institutions are divided into formal institutions (e.g., policy documents, laws, and regulations) and informal institutions (e.g., social trust, customs, culture, values), which interact with each other to constrain and shape firm behavior. In the field of international business, several recent studies have begun to focus on the joint effect of formal and informal institutions on international financial contracts (Brockman et al., 2020), foreign direct investment (Zhang, 2022), and staffing strategies (Gaur et al., 2022). Formal and informal institutions are interdependent, exist in all layers of society (Williamson, 2000), and function in concert with each other (North, 1990). North (1990) argue that “formal rules can complement and increase the effectiveness of informal constraints. They may lower information, monitoring, and enforcement costs and hence make informal constraints possible solutions to more complex exchange.” Accordingly, we expect that strong formal institutions can increase the marginal effect of social trust on the location choices of foreign firms.

On the one hand, trust is based on the development of formal institutions, and strong formal institutions help to product high level social trust (Cui, 2017). As Knack and Keefer (1997) argue, strong formal institutions provide stable external safeguards for transactional behavior and help reduce the risk of opportunistic behavior, which can promote the generation of high-level social trust. On the other hand, strong formal institutions help social trust work and can enhance the effectiveness of social trust (Gaur et al., 2022). In regions where formal institutions are strong, firms have higher costs of violation, and firms tend to implement honest and trustworthy behavior and are less likely to implement opportunistic behavior. In such cases, formal institutions and social trust have complementary effects in influencing foreign firms’ locational choices. This hypothesis is consistent with previous studies, such as Child and Möllering (2003) found that the trust of parent country nationals (PCNs) in host country nationals (PCNs) is further strengthened by contextual confidence in formal institutions that support trusting relations. Moreover, Gaur et al. (2022) found that efficient and fair formal institutions form the basis for developing social trust by signaling that untrustworthy behavior is sanctioned, jointly reduce the incidence of expatriate staffing in foreign subsidiaries. In summary, regions with strong formal institutions are conducive to the generation and functioning of social trust, which provides a stable investment and business environment for foreign firms, and thus promotes the location choices of foreign firms. Based on the foregoing we propose:

Hypothesis 2: Formal institution moderates positively the relationship between social trust and the location choices of foreign firms in subnational regions.

### 3.3. The moderating role of cultural distance

“International management is the management of distance”(Zaheer et al., 2012). The concept of cultural distance is widely used in the field of international business (Bi, 2017). Some literature in the field of international business has explored the effect of home and host country distances, especially cultural distances, on MNEs’ location choices for overseas investments (Du et al., 2012; Bailey and Li, 2015), since overseas investments inevitably involve exchanges between different culture. Transaction cost theory suggests that the greater the cultural distance between the host and home countries, the higher the transaction costs and uncertainty of foreign firms, which diminishes the willingness of MNEs’ investments. According to the previous theoretical analysis, we can know that social trust helps to reduce the costs and uncertainty of foreign firms operating in the host country. We expect that the greater the cultural distance, social trust can play a more important role in helping foreign firms to gain legitimacy in the host country (Arslan and Dikova, 2015). Specifically, the increased cultural distance not only makes it more difficult for foreign firms to understand host country laws and regulations and adapt to local social customs (Ang et al., 2015), but also makes it difficult for foreign firms to gain the favor of host country governments, suppliers, customers, and communities (Yi et al., 2019). In such cases, the trust of members of the host society in foreign firms can play a greater role in helping foreign firms to integrate into the social network and reduce uncertainty and costs. Conversely, in host countries with less cultural distance, there are fewer obstacles to communication and coordination between foreign and local firms. Communication and cooperation with high efficiency between the two parties is easier, so foreign firms can quickly adapt to the market, institutions, and business environment (Du et al., 2012). In such cases, foreign firms face lower uncertainty and transaction costs, and social trust plays a weaker role in influencing the location choices of foreign firms. In summary, the greater the cultural distance between the host and home countries, the greater the role of social trust in reducing the uncertainty and costs, thus having a greater effect on foreign firms’ location choices. Thus, the following hypothesis is proposed:

Hypothesis 3: The cultural distance between the home and host country moderates positively the relationship between social trust and the location choices of foreign firms in subnational regions.

## 4. Research design

### 4.1. Data and sample

How to obtain data on foreign firms in China is the key and difficult problem of this study. Existing studies on the location choice of foreign firms in China mainly use province-level or city- level data, while a small number of firm-level studies mainly use the China Industrial Enterprise Database. However, this database only includes industrial firms with sales above 5 million RMB (20 million RMB since 2011), and has the limitation of a short available period (the latest is generally up to 2013). In order to obtain more complete data on foreign firms in China, we constructed a database of foreign firms and their parent companies in China based on the Orbis Global Enterprise Database. Since the location information in the Orbis database is spelled in English or has a small number of missing information, whereas different regions of China may have the same spelling in English, it cannot be used directly. For this reason, we cross checked and manually supplemented by manually finding the names, contact information, registration time and location zip codes of foreign firms, and finally obtained the data related to foreign firms in China from 2008 to 2020.

Other data involved in this study were mainly obtained from databases such as the China General Social Survey (CGSS) database, China’s provincial statistical yearbooks, the Hofstede insights database, and the Marketization Index of China’s provinces. In addition, we performed the following processing. (1) We define foreign firms as the shareholding ratio of foreign parent company is not less than 10%, and therefore exclude samples with less than 10% foreign parent ownership. (2) Considering the special characteristics of finance and insurance industries, we exclude foreign firms in finance and insurance industries. (3) China has preferential policy treatment for foreign firms in many aspects, so some foreign firms may be fake foreign firms. That is, local Chinese firms go to the British Virgin Islands, Cayman Islands, Bermuda or other tax havens to register and then return to China to invest in order to receive preferential policies. Therefore, we exclude foreign firms whose home countries are British Virgin Islands, Cayman Islands, Bermuda or other tax havens. (4) We finally exclude the samples containing missing values in the consolidated data. Finally, we obtained data on 10,045 foreign firms’ investments in China for 31 Chinese provinces from 2008 to 2020, totaling 311,395 observations. To alleviate the disruptions of extreme values on the study results, we performed bilateral tailing at the 1% level for the main continuous variables.

### 4.2. Measurement of variable

#### 4.2.1. Dependent variable

Location choice of foreign firms in China (Location). If the parent company of a foreign firm chooses a province to establish a foreign firm in the year of observation, the province is recorded as 1 and the other provinces are recorded as 0.

#### 4.2.2. Independent variable

Social trust (STL). In recent years, the CGSS database has been used by several scholars to measure social trust in various provinces in China (Lu et al., 2018). The CGSS database provides survey data on social trust across Chinese provinces, “In general, do you agree that in this society, the majority of people can be trusted?” (Strongly disagree = −2, Disagree = −1, Neither agree nor disagree = 0, Agree = 1, Strongly agree = 2), and the social trust (STL) of the region was measured as the average of the respondents in each province. A higher value for a province indicates a higher social trust in that province.

#### 4.2.3. Moderating variable

Formal Institution (INS). We use the Marketization Index of China’s provinces (2018) (Wang et al., 2019) to measure the formal institution, and the missing data are calculated using a continuous 5 years moving average growth rate. Cultural distance (CD). We followed Kogut and Singh (1988) calculation method and used a country’s cultural characteristics including power distance, uncertainty avoidance, individualism and collectivism, masculinity and femininity, long-vs. and short-term orientation to measure the cultural distance. The data were obtained from the official Hofstede Insights website and the calculation formula is shown in equation (1).


(1) $$C\,D_{\text{ic}}=\sum_{j=1}^{n}\left[\frac{{\left(I_{i\,j}-I_{c\,j}\right)}^{2}}{V_{j}}\right]/n$$

Where, *CD*_*ic*_ represents the cultural distance between the host country *i* and China, *j* represents the subdimension, *I*_*ij*_ represents the value of the host country *i* on the dimension *j*, *I*_*cj*_ represents the value of China on the dimension *j*, *V*_*j*_ represents the variance of the dimension *j*, and *n* represents the number of indicators measured.

#### 4.2.4. Control variables

In the conditional logit model, the firm-level factors have the same value in each alternative and their within-group variance is zero, so they cannot be included in the study model (Chung and Alcácer, 2002; Chang and Park, 2005; Belderbos et al., 2011; Tan and Meyer, 2011; Li et al., 2019). Referring to the approach of existing studies (Chung and Alcácer, 2002; Chang and Park, 2005; Belderbos et al., 2011; Tan and Meyer, 2011; Li et al., 2019), we controlled for factors in subnational regions that may influence the location choices of foreign firms. The control variables include GDP per capita (PGDP), population density (Pop), wage of employees in a province (Wage), openness to the outside world (Open), the quality of the labor in each province (Labor), the ratio of tertiary industry value added in GDP (Industry), and the ratio of road area to total population (Infra).

### 4.3. Research model

This study uses a conditional logit model to test the research hypotheses as this model is particularly appropriate in models of choice behavior where the explanatory variables include attributes of the choice alternatives (Kang and Liu, 2016). Moreover, conditional logit model is widely used in studies on foreign subsidiaries’ location choice (Chung and Alcácer, 2002; Chang and Park, 2005; Belderbos et al., 2011; Tan and Meyer, 2011; Li et al., 2019). The conditional logit model is able to predict the probability of achieving a certain outcome (in this study, the “yes” or “no” decision of foreign firms to invest in Chinese provinces) depending on the value or state of the independent variable (social trust) (Kang and Liu, 2016). The principle of the conditional logit model is as follows. Foreign firms planning to enter the Chinese market face the problem of choosing alternative investment locations *j* (*j* = 1, 2,…,*n*). It is assumed that the utility functions of foreign firms investing in different regions of China are as shown in equation (2).


(2) $$U_{\text{ij}}=\beta_{1}\,S\,T\,L_{i\,j}+\gamma \,x_{i\,j}+\varepsilon_{i\,j}$$

Where, *i* represents foreign firms and *j* represents the regions that foreign firms can choose to invest in China. *STL*_*ij*_ represents the level of social trust in region *j* that foreign firm *i*can choose. *x*_*ij*_ represents the other characteristics (control variables: GDP per capita, population density, wage of employees in a province, openness to the outside world, the quality of the labor in each province, the ratio of tertiary industry value added in GDP and the ratio of road area to total population) of region *j* that foreign firms *i* can choose, and ε_*ij*_ is a stochastic error term. If a foreign firm chooses region *j* among the *n* alternative regions and assumes that this choice will maximize the firm’s utility, then the foreign firm needs to satisfy that the utility of investing in region *j* is greater than the utility of investing in region *k*, as shown in equation (3).


(3) $$U_{i\,j}>U_{i\,k}\,\forall j\neq k$$

The utility function *U*_*ij*_ of foreign firms in alternative regions cannot be observed directly, but can only be expressed by observing the regions in which foreign firms actually invest. The probability of foreign firms investing in region *j* is shown in equation (4).


(4) $$P\left(L\text{o}cation_{i\,j}=1\right)=\frac{\exp\left(\beta_{1}\,S\,T\,L_{i\,j}+\gamma \,x_{i\,j}\right)}{\sum_{j=1}^{n}\exp\left(\beta_{1}\,S\,T\,L_{i\,j}+\gamma \,x_{i\,j}\right)}$$

where the explanatory variable *Location*_*ij*_ indicates whether foreign firm *i* invests in region *j* among the *n* alternative regions, and the value of *Location*_*ij*_ is 1 when the foreign firm *i* invests in region *j* and 0 otherwise.

## 5. Results

### 5.1. Descriptive statistics

Table 1 reports the results of descriptive statistics and correlation analysis. The mean value of social trust is 0.470 and the standard deviation is 0.251, indicating significant differences in social trust between subnational regions in China. The results of the correlation analysis show that the correlation coefficient is less than the recommended value of 0.8 (Fornell and Larcker, 1981), but there are several variables that are highly correlated, which is determined by the way the sample is constructed. That is, because a small number of firms and location attributes are repeated in different selection sets (not uncommon in datasets with repeated observations) (Li et al., 2019). PGDP is highly correlated with many variables (especially Wage and Industry) because it is an umbrella indicator of economic health that reflects the general attractiveness of a region. We retested our model after excluding PGDP or other highly correlated variables, following the practice of scholars (Li et al., 2019; Gaur et al., 2022), and found no qualitative change in the results. In addition, the variance inflation factor results showed that the highest value of VIF was 7.30 and the mean value was 3.15, both of them are below the suggested threshold of 10 (Chatterjee and Price, 1991). Therefore, multicollinearity is not concern for the results of our study.

**TABLE 1:** Descriptive variable statistics.

	1	2	3	4	5	6	7	8	9	10
1. STL	1									
2. INS	**0**.**219**	1								
3. CD	**0**.**009**	0.001	1							
4. PGDP	**0**.**103**	**0**.**655**	-0.002	1						
5. Pop	**0**.**082**	**0**.**025**	-0.000	**−0**.**101**	1					
6. Wage	**0**.**033**	**0**.**260**	**−0**.**014**	**0**.**742**	**−0**.**050**	1				
7. Open	**−0**.**105**	**0**.**582**	0.001	**0**.**623**	**−0**.**113**	**0**.**196**	1			
8. Labor	**0**.**125**	**0**.**496**	-0.002	**0**.**577**	**0**.**083**	**0**.**376**	**0**.**442**	1		
9. Industry	**0**.**045**	**0**.**191**	0.000	**0**.**701**	**−0**.**245**	**0**.**719**	**0**.**496**	**0**.**449**	1	
10. Infra	**0**.**070**	**0**.**026**	-0.001	**−0**.**042**	**−0**.**236**	**0**.**090**	**−0**.**337**	**−0**.**178**	**−0**.**241**	1
Mean	0.470	7.648	2.536	4.980	0.281	6.309	0.042	1.176	48.481	15.393
S.D.	0.251	2.108	1.059	2.656	0.115	3.008	0.045	0.443	9.060	4.705
VIF	1.28	3.31	1.00	7.30	1.43	5.13	3.61	1.72	5.03	1.64

*N* = 311395, numbers in bold indicate statistical significance at the 0.01 level.

### 5.2. Empirical regression results

The regression results of this study are shown in Table 2. Model (1) shows that the estimated coefficient of the core explanatory variable STL is significantly positive (β = 0.209, *p* < 0.05) after controlling the subnational region variable. This indicates that the region with higher level social trust, the foreign firms are more likely to invest in such region, and hypothesis 1 is supported. Model (2) shows the regression results of the moderating effect of formal institutions, and the coefficient of the interaction term between social trust and formal institutions (STL × INS) is significantly positive (β = 0.313, *p* < 0.001). This indicates that the region with stronger formal institutions, the positive effect of social trust on foreign firms’ investment is greater, and hypothesis 2 is supported. Model (3) shows the regression results of the moderating effect of cultural distance, and the coefficient of the social trust and cultural distance interaction term (STL × CD) is significantly positive (β = 0.137, *p* < 0.05). This indicates that the cultural distance between home and host country is higher, the positive effect of social trust on foreign firms’ investment is greater, and hypothesis 3 is supported.

**TABLE 2:** Regression results of conditional logit model.

	Model (1)	Model (2)	Model (3)
STL	0.209** (0.090)	−3.079*** (0.596)	−0.136 (0.191)
INS		0.080** (0.036)	
STL × INS		0.313*** (0.057)	
CD			0.000 (.)
STL × CD			0.137** (0.067)
PGDP	0.073*** (0.025)	0.064** (0.026)	0.073*** (0.025)
Pop	−0.564 (0.505)	−0.310 (0.507)	−0.562 (0.505)
Wage	0.120*** (0.027)	0.060** (0.028)	0.120*** (0.027)
Open	1.814* (1.086)	0.465 (1.095)	1.813* (1.086)
Labor	0.337 (0.259)	−0.130 (0.266)	0.337 (0.259)
Industry	0.025*** (0.007)	0.023*** (0.007)	0.025*** (0.007)
Infra	−0.006 (0.015)	−0.001 (0.015)	−0.006 (0.015)
*N*	311395	311395	311395
pseudo *R*^2^	0.341	0.342	0.341
Log likelihood	−22734.726	−22692.099	−22732.636

*p < 0.1. **p < 0.05. ***p < 0.01. Standard errors in parentheses. Since for each foreign firm, the value of CD is the same and the variance within the CD group is 0, it is excluded from the model, as below.

### 5.3. Robustness tests

#### 5.3.1. Testing the IIA hypothesis

An important prerequisite for using conditional logit models is to satisfy the IIA hypothesis. We test the IIA hypothesis using the Hausman test (Hausman and McFadden, 1984; Li et al., 2019). The test was performed as follows: by comparing whether there is a systematic difference in the estimated parameters after randomly excluding an alternative province. The null hypothesis was that the IIA hypothesis holds and there is no systematic difference in the estimated results after excluding an alternative province. The test results show that for the sample data in this study, the majority of the chi-square values are very small or negative, which means that no significant difference between the parameters of the restricted and unrestricted models, indicating the IIA hypothesis is satisfied.

#### 5.3.2. Change the indicator of the independent variable

Since social trust is the key variable in this study, we conducted robustness tests by changing the indicator of social trust. We used the trust index from the data of the trust environment survey of Chinese provinces (Zhang and Ke, 2002) as a proxy variable for social trust. The regression results are shown in Table 3. After replacing the social trust indicator, the conclusions are consistent with the previous results and the findings remain robust.

**TABLE 3:** Regression results of changing independent variable indicator.

	Model (1)	Model (2)	Model (3)
Trust index	1.352*** (0.216)	0.129 (0.384)	1.285*** (0.219)
INS		0.056 (0.055)	
Trust index × INS		0.145*** (0.044)	
CD			0.000 (.)
Trust index × CD			0.027** (0.014)
PGDP	0.073*** (0.025)	0.060** (0.027)	0.072*** (0.025)
Pop	−0.540 (0.505)	−0.550 (0.509)	−0.529 (0.505)
Wage	0.116*** (0.027)	0.061** (0.028)	0.118*** (0.027)
Open	1.513 (1.079)	1.366 (1.092)	1.514 (1.079)
Labor	0.321 (0.258)	0.390 (0.273)	0.324 (0.259)
Industry	0.026*** (0.007)	0.010 (0.008)	0.026*** (0.007)
Infra	−0.013 (0.015)	0.002 (0.015)	−0.012 (0.015)
*N*	311395	311395	311395
pseudo *R*^2^	0.341	0.342	0.341
Log likelihood	−22737.460	−22704.266	−22735.530

*p < 0.1. **p < 0.05. ***p < 0.01. Standard errors in parentheses.

#### 5.3.3. Defined thresholds for the replacement of foreign firm

In the previous section, we define foreign firms as the shareholding ratio of foreign parent company is not less than 10%. And some studies use other thresholds to define foreign firms. For this reason, with reference to the Law of the People’s Republic of China on Chinese-Foreign Joint Ventures, we redefine foreign firms as the shareholding ratio of foreign parent company is not less than 25% in the robustness test. The results are shown in Table 4. After replacing the definition thresholds of foreign firms, the conclusions are consistent with the previous study, meaning the results remain robust.

**TABLE 4:** Regression results for replacing the foreign firm definition threshold.

	Model (1)	Model (2)	Model (3)
STL	0.159* (0.092)	−3.185*** (0.618)	−0.205 (0.196)
INS		0.103*** (0.038)	
STL × INS		0.317*** (0.059)	
CD			0.000 (.)
STL × CD			0.145** (0.069)
PGDP	0.071*** (0.026)	0.064** (0.027)	0.071*** (0.026)
Pop	−0.740 (0.522)	−0.490 (0.524)	−0.739 (0.522)
Wage	0.120*** (0.028)	0.054* (0.029)	0.120*** (0.028)
Open	1.483 (1.117)	0.016 (1.126)	1.483 (1.117)
Labor	0.355 (0.266)	−0.151 (0.274)	0.355 (0.266)
Industry	0.027*** (0.007)	0.025*** (0.008)	0.027*** (0.007)
Infra	−0.014 (0.016)	−0.010 (0.016)	−0.014 (0.016)
*N*	295337	295337	295337
pseudo *R*^2^	0.346	0.348	0.346
Log likelihood	−21386.212	−21339.393	−21384.003

*p < 0.1. **p < 0.05. ***p < 0.01. Standard errors in parentheses.

#### 5.3.4. Endogeneity test

The findings of this study regarding social trust facilitating investment of foreign firms may be plagued by endogeneity issues. First, social trust changes slowly, thus firm-level outcomes (locational choice) are unlikely to affect the informal institutions (social trust) in a region (Williamson, 2000), so reverse causality is unlikely to fundamentally change our findings. Second, all variables are measured from well-established measures that are widely used in prior studies, so that measurement error does not significantly affect the results. However, even though this study controls for other factors related to location choices of foreign firms as much as possible, there may still be unobservable variables that affect both social trust and location choices of foreign firms, thus it is impossible to completely resolve the issue of omitted variables which could lead to endogeneity. Therefore, we use the instrumental variables approach to estimate the true effect of social trust on location choices of foreign firms.

Based on existing research (Lu et al., 2018), we use ethnic diversity (ED) as an instrumental variable for endogeneity testing. On the one hand, Dinesen and Sønderskov (2015) found that ethnic diversity has a negative effect on social trust because exposure to people with different ethnic background “spurs conflict and competition over scarce resources,” thus relevance is satisfied. On the other hand, ethnic diversity is mainly determined by historical factors (Lu et al., 2018) and is not related to the location choices of foreign firms, so exogeneity is also satisfied. In summary, ethnic diversity is negatively correlated with social trust and is not correlated with foreign firms’ location choice. So ethnic diversity can be used as a negative instrumental variable of social trust to test the robustness of the conclusions under the omitted variable problem.

Since the conditional logit model fails in two-stage least squares, we refer to Hilbe (2011) and use the number of foreign firms in each province (Quantity) as a proxy variable for foreign firms’ location choice, followed by regression tests using Poisson regression, Negative Binomial regression and Negative Binomial regression instrumental variable two-step method (Hilbe, 2011). The steps are as follows.

First, we perform Poisson regression and Negative Binomial regression tests on the relationship between social trust and the number of foreign firms in each province, considering the time and region dimensions, and the results are shown in Table 5. Model (1) shows the result of Poisson regression, and the coefficient of social trust is significantly positive, indicating that social trust positively influences the location choices of foreign firms (*p* < 0.01). Model (2) shows the result of Negative Binomial regression, and the log-likelihood value is significantly higher than Poisson regression, which indicates that using Negative Binomial regression is more effective. The results for the first stage in Model (3) suggest that the ED has a significant negative effect on social trust (*p* < 0.01), consistent with the findings from prior studies (Dinesen and Sønderskov, 2015), and the first-stage F value is greater than 10 which indicates that there is no weak instrumental variable problem. The results for the second stage in Model (4) show that, after solving the omitted variable problem, social trust still has a positive and significant effect on the location choice of foreign firms (*p* < 0.01), indicating that endogeneity is not a serious threat.

**TABLE 5:** Regression results of the instrumental variable method.

	Model (1)	Model (2)	Model (3)	Model (4)
	Position	Nbreg	OLS	Twostep-Nbreg
STL	0.386*** (0.074)	0.897*** (0.258)		7.051*** (1.016)
ED			−0.385*** (0.110)	
Rhoz				−6.725*** (1.071)
PGDP	0.242*** (0.013)	0.390*** (0.056)	0.042*** (0.009)	0.055 (0.074)
Pop	−0.853*** (0.194)	−3.290*** (0.499)	0.073 (0.126)	−4.781*** (0.539)
Wage	0.132*** (0.016)	−0.110 (0.076)	−0.068*** (0.012)	0.351*** (0.101)
Open	18.229*** (0.445)	21.192*** (1.711)	−1.847*** (0.298)	32.121*** (2.428)
Labor	−0.458*** (0.037)	0.062 (0.130)	0.035 (0.027)	−0.278** (0.132)
Industry	−0.054*** (0.003)	−0.102*** (0.012)	0.007*** (0.002)	−0.137*** (0.013)
Infra	0.049*** (0.004)	−0.028** (0.013)	−0.000 (0.003)	−0.040*** (0.013)
Area	Yes	Yes	Yes	Yes
Time	Yes	Yes	Yes	Yes
*N*	403	403	403	403
pseudo *R*^2^*/R*^2^	0.842	0.219	0.376	0.232
F			11.966	
Log likelihood	−2225.379	−1171.524	101.696	−1151.511

*p < 0.1. **p < 0.05. ***p < 0.01. Standard errors in parentheses.

### 5.4. Analysis of effect mechanisms

According to the previous theoretical analysis, promoting information flow, reducing transaction costs and improving innovation efficiency are important mechanisms for social trust to affect the location choices of foreign firms. Since the three-step mediation mechanism test (Baron and Kenny, 1986) fails in the conditional logit model, we take the number of foreign firms attracted by each province as the dependent variable and use Negative Binomial regression model to test the three mentioned mediating mechanisms.

#### 5.4.1. Cost advantage mechanism test

Theoretically, foreign firms need to consider the level of regional financing constraints when choosing an overseas investment location, because high financing costs imply a corresponding increase in the firm’s transaction costs. Therefore, we use the financing constraint at the provincial-level as a proxy of the cost advantage. We use the provincial financial institution network density (finance) to indicate the difficulty of regional financing constraints (Zhao et al., 2021), and then test the cost advantage mechanism. The results are shown in Table 6. The foreign firms’ location choices (Quantity) were first regressed on social trust (STL), it was found from Model (1) that STL had a positive significant effect on foreign firm location choices (*p* < 0.01). Model (2) shows the regression results between social trust and finance. The higher density of financial institutions’ network represents lower transaction cost, so the results of Model (2) indicate that social trust helps to reduce firms’ transaction costs (*p* < 0.01). And when both finance and social trust were entered into the Model 3, the effects of STL on foreign firms’ location choice were weakened, and remained significant (*p* < 0.01). The effects of cost advantage were still significant (*p* < 0.1). According to Baron and Kenny (1986), this supports the mediation effect of cost advantage on the relationship between STL and the location choices of foreign firms.

**TABLE 6:** Regression results for the intermediary mechanisms.

	Model (1)	Model (2)	Model (3)	Model (4)	Model (5)	Model (6)	Model (7)
	Quantity	Finance	Quantity	Information	Quantity	Innovation	Quantity
STL	0.878***	0.387***	0.802***	0.309**	0.467**	0.708***	0.809***
	(0.255)	(0.135)	(0.258)	(0.121)	(0.234)	(0.209)	(0.247)
Finance			0.015*				
			(0.008)				
Information					0.046***		
					(0.004)		
Innovation							0.051***
							(0.009)
PGDP	0.384***	0.072***	0.377***	0.203***	0.158***	0.280***	0.233***
	(0.056)	(0.022)	(0.056)	(0.027)	(0.048)	(0.041)	(0.057)
Pop	-3.332***	0.635**	-3.517***	-0.639**	-3.057***	-0.790*	-3.334***
	(0.494)	(0.254)	(0.504)	(0.268)	(0.428)	(0.452)	(0.471)
Wage	-0.125*	0.100***	-0.173**	-0.360***	0.197***	-0.262***	-0.019
	(0.076)	(0.028)	(0.082)	(0.039)	(0.065)	(0.061)	(0.071)
Open	21.344***	7.767***	20.637***	8.615***	15.301***	14.086***	18.212***
	(1.680)	(0.713)	(1.700)	(0.952)	(1.476)	(1.342)	(1.664)
Labor	0.087	0.425***	0.077	-0.497***	0.856***	-0.560***	0.443***
	(0.129)	(0.058)	(0.129)	(0.077)	(0.126)	(0.112)	(0.136)
Industry	-0.100***	-0.019***	-0.100***	-0.024***	-0.093***	-0.032***	-0.092***
	(0.012)	(0.005)	(0.012)	(0.006)	(0.010)	(0.010)	(0.011)
Infra	-0.024*	-0.012*	-0.017	-0.017**	-0.033***	0.013	-0.037***
	(0.013)	(0.006)	(0.014)	(0.008)	(0.011)	(0.012)	(0.013)
Area	Yes	Yes	Yes	Yes	Yes	Yes	Yes
Time	Yes	Yes	Yes	Yes	Yes	Yes	Yes
*N*	403	403	403	403	403	403	403
pseudo *R*^2^	0.217	0.315	0.218	0.203	0.258	0.271	0.228
Log likelihood	-1167.334	-841.078	-1165.737	-1223.030	-1106.254	-763.691	-1151.109

*p < 0.1. **p < 0.05. ***p < 0.01. Standard errors in parentheses.

#### 5.4.2. Information advantage mechanism test

In today’s economic activities, internet infrastructure development can reduce the costs of information search and access, facilitate the flow and sharing of information, and alleviate information asymmetry (Meijers, 2013). Therefore, we use the number of internet access ports (information) as a measure of information advantage (Meijers, 2013) and then test the information advantage mechanism. The results are shown in Table 6. The information was regressed on social trust (STL), it was found from Model (4) that STL had a positive significant effect on information (*p* < 0.05). And when both information and social trust were entered into the Model (5), the effects of STL on foreign firms’ location choice were weakened, and remained significant (*p* < 0.05). The effects of information advantage were still significant (*p* < 0.01). According to Baron and Kenny (1986), this supports the mediation effect of information advantage on the relationship between STL and the location choices of foreign firms.

#### 5.4.3. Innovation advantage mechanism test

A region with high patent output and efficient innovation has a great attractiveness to the location choices of foreign firms. In order to ascertain whether innovation advantage is a mediator, we choose the number of patents granted (innovation) to measure regional innovation efficiency (Krammer, 2009) and then test the innovation advantage mechanism. The results are presented in Table 6. The innovation was regressed on social trust (STL), it was found from Model (6) that STL had a positive significant effect on innovation (*p* < 0.01). And when both innovation and social trust were entered into the Model (7), the effects of STL on foreign firms’ location choice were weakened, and remained significant (*p* < 0.01). The effects of innovation advantage were still significant (*p* < 0.01). According to Baron and Kenny (1986), this supports the mediation effect of innovation advantage on the relationship between STL and the location choices of foreign firms.

## 6. Conclusion and discussion

### 6.1. Conclusion

Based on institutional theory and the Chinese context, this study examines the effect and boundary conditions of social trust on the location of foreign firms using the Orbis Global Enterprise Database. In this paper, there are several main findings.

Firstly, social trust is positively related to the location choices of foreign firms in subnational regions. This finding is consistent with our expected hypothesis. That is, social trust, as an important informal institution, can provide a favorable institutional environment for foreign firms to operate and reduce foreign business uncertainty and attract foreign firms’ investment through cost advantage, information advantage, and innovation advantage. Previous studies have shown that social trust, as an important dimension of social capital, plays an important role in enhancing the authenticity and reliability of information shared by members of society (Jin et al., 2016; Jha, 2019), promoting and maintaining cooperative relationships (Porta et al., 1997; Constantin et al., 2013), and stimulating innovation within and among organizations (Quinn, 1979). In the field of international business, the impact of social trust is very broad. Social trust not only helps to facilitate OFDI and trade transactions between countries (Guiso et al., 2009), but also has important implications for both the internationalization strategies and performance of firms (Lu et al., 2018; Gaur et al., 2022). In addition, foreign firms operating in an institutional environment different from their home country need to consider their investment location choices for different regions within the country (Du et al., 2008). However, the existing literature is more based on the country level to study the impact of institutional differences in different countries (Brockman et al., 2020; Gaur et al., 2022). In our study, social trust in each province shapes the external environment in which foreign firms operate in China and has a potential impact on the risks and benefits of foreign firms’ operations in China, which in turn affects their locational choices. Our study develops a stream of research not only on the impact of social trust on multinational firms’ internationalization strategies (location choice), but also on the impact of subnational institutions on firms’ internationalization strategies.

In addition, this study suggests that the effect of social trust on location choices of foreign firms is not homogenous. The findings clearly indicate that the relative strength of social trust’s effect on location choice of foreign firms depends on country-level and regional-level factors. Specifically, social trust will have a stronger effect on location choice of foreign firms in regions with better-developed formal institutions, for the higher cultural distance between home and host country. As North (1990) argues, “formal rules can complement and increase the effective-ness of informal constraints. They may lower information, monitoring, and enforcement costs and hence make informal constraints possible solutions to more complex exchange.” Thus, well-established formal institutions provide a stable external guarantee for economic activities, which can complement and increase the effectiveness of constraints of social trust. Besides, social trust will have a stronger effect on location choice of foreign firms when the home and host country with higher cultural distance. The rationale is that higher cultural distance not only makes it more difficult for foreign firms to understand the institutions of host country (Ang et al., 2015), but also makes it difficult for foreign firms to access information and resources in the host country (Yi et al., 2019). At this time, trust in foreign firms by members of the host society can play a greater role in helping foreign firms integrate into the host society network and reduce uncertainty and operating costs.

### 6.2. Theoretical contributions

This study makes theoretical contributes to the location choice, institutional theory and social trust literature in several ways. Firstly, this study makes contributions to the location choice of foreign firm literature. Location choice of foreign firm, subsumed under a broader foreign market entry strategy, is an important decision for MNE. IB scholars have accumulated rich discussions on the factors influencing location choice strategy from different theoretical perspectives. Based on institutional theory, this study adds to this research, highlighting the effect of social trust (an important part of informal institutions) on the location choice, helping us to understand the economic effect of the “soft environment” of social trust. Thus, this study complements previous studies’ focus on economic factors such as labor costs, infrastructure, market size and exchange rates (Dunning, 1980; Grosse and Trevino, 1996) and formal institutions such as the level of corruption (Brada et al., 2012), host country governance (Louie and Rousslang, 2007) or tax rates (Davies and Killeen, 2018).

Secondly, different from previous studies that have taken the country as the unit of analysis to examine the effect of cross-country institutional differences on the location choice of foreign firms, our study examines the impact of intra-country subnational institutional complexity on the location choice of foreign firms. As Beugelsdijk et al. (2010) showed, IB scholars tend to regard “location” as being the synonymous with “country,” and often assume country or national borders as the unit of analyses. However, such dominant focus on cross-country variations can be incomplete and sometimes even problematic (Chan and Du, 2022), because “the types of subnational spatial variation ignored by analyses based on country averages are precisely what shape firm location strategies” (Beugelsdijk and Mudambi, 2013). In this study, we focus on institutional differences within host countries and examine how MNCs make locational choices of foreign subsidiaries in specific institutional environments at the subnational level. In addition, cross-country studies are likely to confound many factors. In contrast, our analysis of single country regional institutions allows us to keep many constant aspects such as culture and language, national tax policies, exchange rates, and trade policies, which may vary significantly across countries (Du et al., 2012). Therefore, our study of a single country is more favorable than cross-country studies in capturing the influence of institutional factors.

Thirdly, this study makes contributions to the institutional theory literature. In the 1990s, following the influential work of North (1990), IB researchers began to pay more attention on the influence of institutions on the location choice of FDI (Bailey, 2018). However, research has mainly focused on the independent effects of specific institutional factors, while less research has been conducted on the interdependence between different institutions. The existing literature on individual institutional factors limits our understanding of the complexity arising from the interactions between different institutional elements (Dau et al., 2020). Some studies have explored the substitution effects of formal institutions and informal institutions. However, institutional interactions lead to multiple configurations, and the interactions go beyond substitution, which can be contradictory or complementary (Helmke and Levitsky, 2004; Fiori, 2018). Our study finds that formal institutions enhance the effectiveness of informal institutional constraints, based on an institutional interaction framework, which helps us to better understand the interaction effects of formal and informal institutions in the location choices of foreign firms. Thus, Consistent with Chan and Du (2022) and Gaur et al. (2022), our study helps to promote a more interactive institution-based perspective.

Finally, this study contributes to the social trust literature. IB studies have shown that social trust affects entry modes (Jin et al., 2016) and performance (Lu et al., 2018) by facilitating information dissemination and reducing transaction costs. This study finds that social trust helps increase the attractiveness of FDI in subnational regions, highlighting the role of social trust in helping foreign firms reduce the uncertainty of overseas investment, which enriches the research related to social trust and its role effects. In addition, this paper takes cultural distance between home and host countries as an important contextual variable, and then investigates the effect of social trust on location choice strategies under different cultural distance contexts. The rationale is that with the deepening of economic globalization, the heterogeneity of countries will become more obvious (Nielsen et al., 2017). In other words, country distance is a non-negligible factor that affects the location choice of MNEs’ overseas investments. The literature on the influence of cultural distance on location choice supports the view that the greater the cultural distance, the more difficult it is for foreign firms to operate, and therefore the lower the likelihood to receive FDI from MENs (Bailey and Li, 2015; Yao and Li, 2016). Moreover, although a large number of studies focus on the effect of cultural distance on location choice, they mostly study the direct effect of cultural distance (Flores and Aguilera, 2007; Yao and Li, 2016), and fewer studies consider it as an important contextual variable and thus study the effect of the subnational regional regime of the host country on location choice in different cultural distance contexts. Similar to Liu et al. (2022) have found that higher cultural tolerance and trust (informal institutions) can facilitate outward FDI by Chinese firms in high cultural distance host countries. This study suggest that subnational social trust helps regions of China to attract investment from firms in countries with higher cultural distance, highlighting the important role of social trust in attracting FDI.

### 6.3. Practical contributions

Our findings have important practical implications for government policymakers and managers of MNEs. Firstly, for government policymakers, they should actively advocate the value of honesty and trustworthiness, implement strict incentives and penalties for trustworthy and untrustworthy behavior, as social trust is an important factor in attracting foreign direct investment. What’s more, we find that formal institutions enhance the role of social trust in attracting foreign investment, so policymakers need to further improve the formal institutional such as laws and regulations to better exploit the complementary effect of formal institutions and social trust in attracting foreign investment. Secondly, Emerging economies have huge growth potential and represent lucrative markets that MNEs cannot ignore. In order to capture profitable market opportunities, MNEs must find ways to deal with the challenging institutional environment in emerging economies. This study finds that high levels of social trust help foreign firms access local market information and resources that they need to succeed and survive in emerging economies. Therefore, for managers of MNEs, especially those with a large cultural distance between their home and host countries, they must realize that high level social trust plays an important role in reducing entry barriers into a region, which is very critical to foreign firms. Thus, all things being equal, foreign firms should choose regions with higher level social trust to benefit from local information advantages, cost advantages and innovation advantages. If they have to enter a region with lower level of social trust, foreign firms should seek local companies as partners to mitigate the negative impact of low social trust. In addition, foreign firms need realize the positive effect of formal institutions on social trust and take formal institution as an important reference factor for location selection. Finally, MNEs should be aware that the institutional environment varies greatly not only among countries, but also in host country sub-national regions, especially in emerging economies such as China. Given that location choice has a profound impact on the survival and profitability of foreign subsidiaries, MNEs should have a holistic view of not only the institutional environment of the host country but also a thorough understanding of the “rules of the games” in the subnational region when formulating location choice strategies.

### 6.4. Limitations and future research

This study has some limitations that could also provide possibilities for further research. Firstly, the findings only relate to Chinese foreign direct investment, and the results may be biased by country specific variables, so the findings may not be fully applicable to other countries. Although China represents an important emerging economy and provides a good research context for this study. Future research could explore the effect of subnational institutions on locational choice in other emerging or developed economies, where the number and quality of institutions may differ. Secondly, due to data limitations, we were unable to distinguish between the in-group and out-group dimensions of social trust (Delhey et al., 2011). They may have different effects on the location choice of foreign firms, it would be important to collect data in this area and examine their varying influence on the location choices of foreign firms. Thirdly, this study only explores the effect of subnational regional social trust on foreign firms’ location choice, and the effect of subnational regional social trust on the economic consequences of foreign firms’ such as entry mode and innovation can be further explored in the future.

## Data availability statement

Publicly available datasets were analyzed in this study. This data can be found at: the Orbis Global Enterprise Database (https://login.bvdinfo.com), the China General Social Survey (CGSS) database (http://cgss.ruc.edu.cn), China’s provincial statistical yearbooks (https://db.cei.cn/jsps/Home), the Hofstede insights database (https://www.hofstede-insights.com), and the Marketization Index of China’s provinces (https://cmi.ssap.com.cn).

## Ethics statement

Ethical review and approval was not required for the study on human participants in accordance with the local legislation and institutional requirements. Written informed consent from the (patients/participants or patients/participants legal guardian/next of kin) was not required to participate in this study in accordance with the national legislation and the institutional requirements.

## Author contributions

HS and CY contributed to conception and design of the study. HS and JY organized the database. HS, JY, and JG performed the statistical analysis. HS wrote the first draft of the manuscript. JY wrote sections of the manuscript. All the authors contributed to manuscript revision, read, and approved the submitted version.
